# Supplementary material for: Behavioural changes in frontotemporal dementia and their cognitive and neuroanatomical correlates
Source: Brain. 2025 Feb 12;148(8):2730–45. doi: 10.1093/brain/awaf061 (PMC12316017; doi:10.1093/brain/awaf061)
Supplement: awaf061_Supplementary_Data [file awaf061_supplementary_data.pdf]

SUPPLEMENTARY TABLES

Supplementary Table 1 Grey matter volumetric differences between groups

Contrast	Hemisphere	Number of voxels	Peak MNI co-ordinate			Peak MNI co-ordinate region	t-value
			x	*y*	*z*		
FTD < Controls							
	Bilateral	263,318	-27	9	-21	Temporal pole	13.53
	Right	3,069	29	-71	-50	Cerebellum	4.97
bvFTD < Controls							
	Bilateral	147,625	2	36	-15	Gyrus rectus	8.94
SD < Controls							
	Bilateral	228,408	-27	3	-23	Temporal pole	21.82
	Right	3,335	29	-69	-48	Cerebellum	4.73
SD < bvFTD							
	Left	9,659	-18	6	-39	Temporal pole	6.23
	Right	2,448	15	-6	-38	Undefined	5,51

bvFTD, behavioural-variant frontotemporal dementia; FTD, frontotemporal dementia; MNI, Montreal Neurological Institute; SD, semantic dementia.

Supplementary Table 2 Percentage of patients impaired on each CBI-R domain

CBI-R Domain	bvFTD	SD	X^2 test
Memory and Orientation (%)	100	100	-
Everyday Skills (%)	96.2	71.4	**$\chi^2 = 5.61$, $P = 0.02$**
Self Care (%)	80.8	23.8	**$\chi^2 = 15.25$, $P < 0.0001$**
Abnormal Behaviour (%)	100	76.2	**$\chi^2 = 6.93$, $P = 0.008$**
Mood (%)	88.5	90.5	$\chi^2 = 0.05$, $P = 0.82$
Beliefs (%)	57,7	33.3	$\chi^2 = 2.77$, $P = 0.1$
Eating Habits (%)	96.2	85.7	$\chi^2 = 1.63$, $P = 0.20$
Sleep (%)	92.3	81.0	$\chi^2 = 1.35$, $P = 0.25$
Stereotypic and Motor Behaviours (%)	92.3	90.5	$\chi^2 = 0.05$, $P = 0.82$
Motivation (%)	100	90.5	$\chi^2 = 2.59$, $P = 0.11$

Significant p-values are highlighted in bold. bvFTD, behavioural-variant frontotemporal dementia; CBI-R, Cambridge Behavioural Inventory-Revised; SD, semantic dementia.

Supplementary Table 3 Percentage of patients impaired on each CBI-R item

Domain	Item	bvFTD	SD	X^2 test
Memory and Orientation (%)	Has poor day-to-day memory	96.2	95.0	$X^2 = 0.04$, $P = 0.85$
Memory and Orientation (%)	Asks the same questions over and over again	88.5	85.7	$X^2 = 0.08$, $P = 0.78$
Memory and Orientation (%)	Loses or misplaces things	92.3	75.0	$X^2 = 2.63$, $P = 0.11$
Memory and Orientation (%)	Forgets the names of familiar people	96.2	95.2	$X^2 = 0.02$, $P = 0.88$
Memory and Orientation (%)	Forgets the names of objects and things	92.0	100	$X^2 = 1.76$, $P = 0.19$
Memory and Orientation (%)	Shows poor concentration when reading or watching television	88.5	81.0	$X^2 = 0.52$, $P = 0.47$
Memory and Orientation (%)	Forgets what day it is	88.5	57.1	**$X^2 = 5.99$, $P = 0.01$**
Memory and Orientation (%)	Becomes confused or muddled in unusual surroundings	92.3	76.2	$X^2 = 2.38$, $P = 0.12$
Everyday Skills (%)	Has difficulties using electrical appliances	88.0	38.1	**$X^2 = 12.52$, $P = 0.0004$**
Everyday Skills (%)	Has difficulties writing letters	92.0	47.6	**$X^2 = 11.09$, $P = 0.0009$**
Everyday Skills (%)	Has difficulties using the telephone	92.0	42.9	**$X^2 = 13.02$, $P = 0.0003$**
Everyday Skills (%)	Has difficulties making a hot drink	48.0	9.5	**$X^2 = 7.98$, $P = 0.005$**
Everyday Skills (%)	Has problems handling money or paying bills	95.8	52.4	**$X^2 = 11.45$, $P = 0.0007$**
Self Care (%)	Has difficulties grooming self	73.1	23.8	**$X^2 = 11.28$, $P = 0.0008$**
Self Care (%)	Has difficulties dressing self	73.1	4.8	**$X^2 = 22.18$, $P < 0.0001$**
Self Care (%)	Has problems feeding self without assistance	26.9	4.8	**$X^2 = 4.04$, $P = 0.04$**
Self Care (%)	Has problems bathing or showering self	64.0	19.0	**$X^2 = 9.39$, $P = 0.002$**
Abnormal Behaviour (%)	Finds humour or laughs at things other do not find funny	92.3	42.9	**$X^2 = 13.58$, $P = 0.0002$**
Abnormal Behaviour (%)	Has temper outbursts	76.9	42.9	**$X^2 = 5.71$, $P = 0.02$**
Abnormal Behaviour (%)	Is uncooperative when asked to do something	96.2	47.6	**$X^2 = 14.39$, $P = 0.0001$**
Abnormal Behaviour (%)	Shows socially embarrassing behaviour	92.0	57.1	**$X^2 = 7.62$, $P = 0.006$**
Abnormal Behaviour (%)	Makes tactless or suggestive remarks	80.8	38.1	**$X^2 = 8.95$, $P = 0.003$**
Abnormal Behaviour (%)	Acts impulsively without thinking	96.2	52.4	**$X^2 = 12.42$, $P = 0.0004$**
Mood (%)	Cries	30.8	33.3	$X^2 = 0.04$, $P = 0.85$
Mood (%)	Mood	66.7	81.0	$X^2 = 1.17$, $P = 0.28$
Mood (%)	Is very restless or agitated	84.0	66.7	$X^2 = 1.89$, $P = 0.17$
Mood (%)	Is very irritable	76.0	47.6	**$X^2 = 3.95$, $P = 0.047$**
Beliefs (%)	Sees things that are not really there	25.0	4.8	$X^2 = 3.27$, $P = 0.07$
Beliefs (%)	Hears voices that are not really there	23.1	0.0	**$X^2 = 5.56$, $P = 0.02$**
Beliefs (%)	Has odd or bizarre ideas that cannot be true	46.2	33.3	$X^2 = 0.79$, $P = 0.37$
Eating Habits (%)	Prefers sweet foods more than before	88.5	76.2	$X^2 = 1.24$, $P = 0.27$
Eating Habits (%)	Wants to eat the same foods repeatedly	80.8	60.0	$X^2 = 2.41$, $P = 0.12$
Eating Habits (%)	Her/his appetite is greater, s/he eats more than before	73.1	23.8	**$X^2 = 11.28$, $P = 0.0008$**
Eating Habits (%)	Table manners are declining	84.6	38.1	**$X^2 = 10.89$, $P = 0.001$**
Sleep (%)	Sleep is disturbed at night	88.5	66.7	$X^2 = 3.30$, $P = 0.07$
Sleep (%)	Sleeps more by day than before	69.2	42.9	$X^2 = 3.31$, $P = 0.07$
Stereotypic and Motor Behaviours (%)	Is rigid and fixed in her/his ideas and opinions	84.6	61.9	$X^2 = 3.15$, $P = 0.08$
Stereotypic and Motor Behaviours (%)	Develops routines from which s/he can not easily be discouraged	76.9	66.7	$X^2 = 0.61$, $P = 0.44$
Stereotypic and Motor Behaviours (%)	Clock watches or appears pre-occupied with time	73.1	57.1	$X^2 = 1.31$, $P = 0.25$
Stereotypic and Motor Behaviours (%)	Repeatedly uses the same expression or catch phrase	80.8	81.0	$X^2 = 0.0003$, $P = 0.99$
Motivation (%)	Shows less enthusiasm for his or her usual interests	88.5	52.4	**$X^2 = 7.56$, $P = 0.006$**
Motivation (%)	Shows little interest in doing new things	92.3	66.7	**$X^2 = 4.93$, $P = 0.03$**
Motivation (%)	Fails to maintain motivation to keep in contact with friends or family	92.3	66.7	**$X^2 = 4.93$, $P = 0.03$**
Motivation (%)	Appears indifferent to the worries and concerns of family members	92.3	61.9	**$X^2 = 6.41$, $P = 0.01$**
Motivation (%)	Shows reduced affection	96.2	57.1	**$X^2 = 10.56$, $P = 0.001$**

Significant p-values are highlighted in bold. bvFTD, behavioural-variant frontotemporal dementia; SD, semantic dementia

Supplementary Table 4 Regions of grey matter intensity correlating with factor scores

	Hemisphere	Number of voxels	Peak MNI co-ordinate			Peak MNI co-ordinate region	*t*-value
			x	*y*	*z*		
PC3 – ADLs							
	Bilateral	11,025	-6	48	14	Anterior cingulate cortex	4.72
	Left	8,871	-29	14	-2	Undefined	4.52
Total Atrophy							
	Bilateral	102,982	-29	20	2	Insula	6.60
	Right	3,792	60	-42	-23	Inferior temporal gyrus	4.58
PC1 – Apathy							
	Bilateral	6,180	2	9	42	Middle cingulate gyrus	5.52

ADLs, activities of daily living; MNI, Montreal Neurological Institute.

SUPPLEMENTARY FIGURES

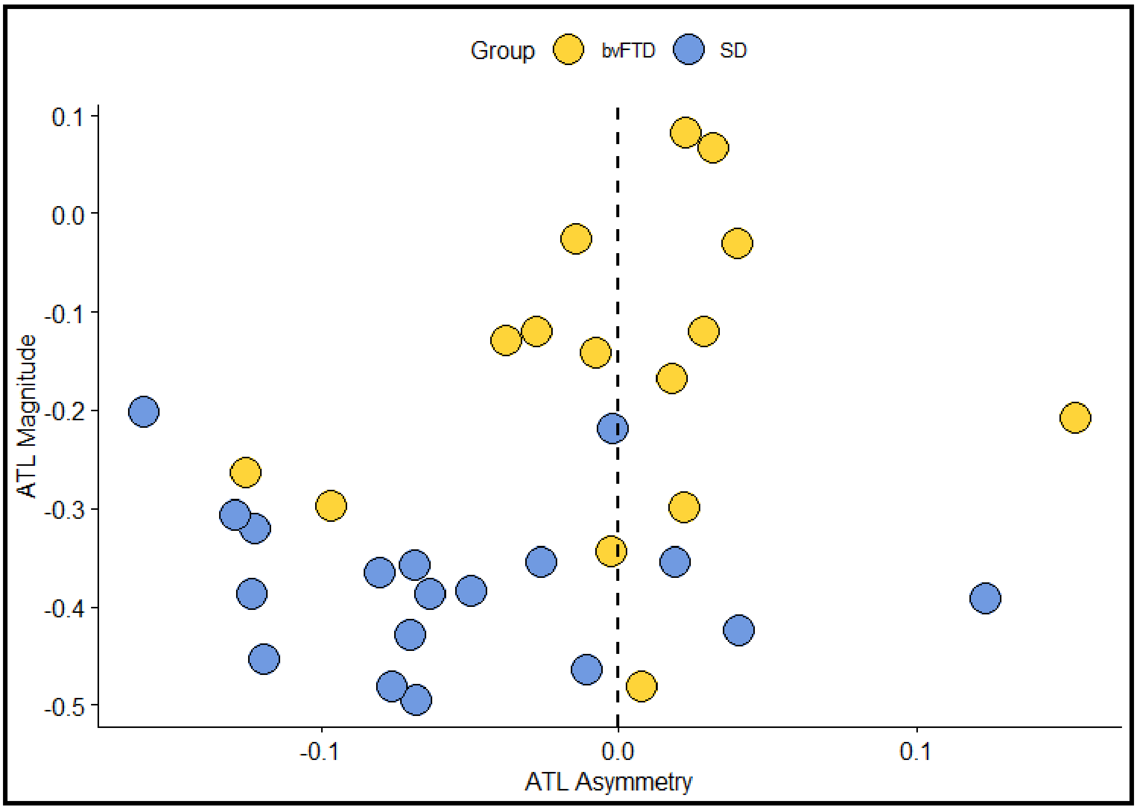

Supplementary Figure 1. ATL indices for each patient. Lower magnitude values indicate increased grey matter volume loss. Negative asymmetry values indicate left>right grey matter volume loss whereas positive asymmetry values indicate right>left grey matter volume loss.

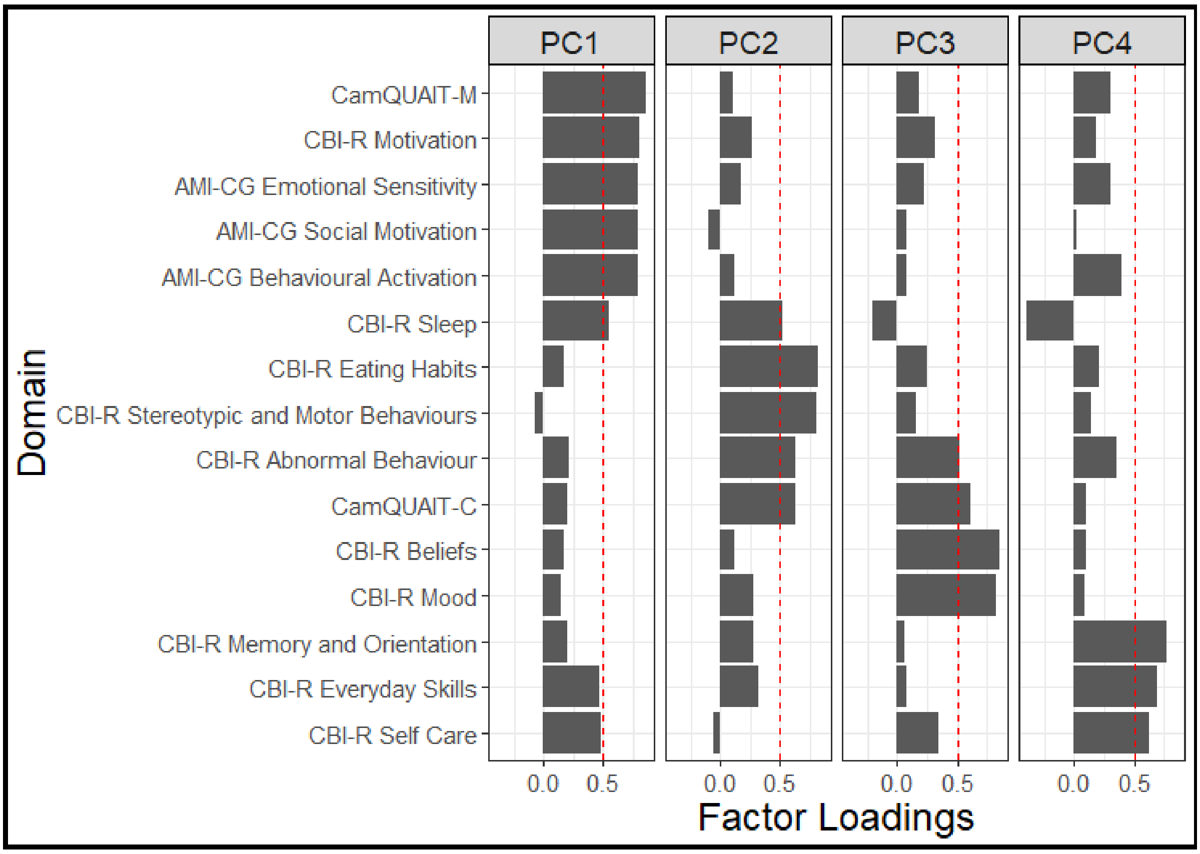

Supplementary Figure 2. **Factor loadings of the informant questionnaire domains for the initial PCA**. Dashed vertical lines indicate factor loading cut-offs (>0.5).

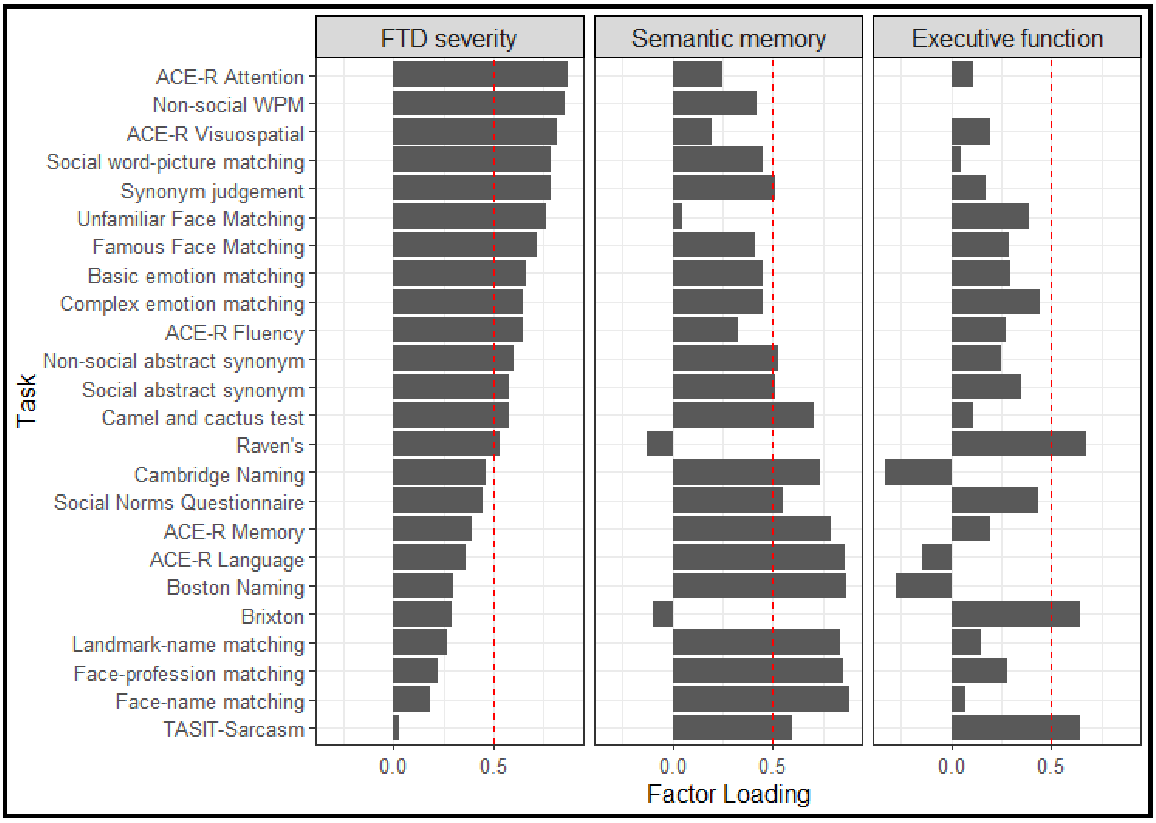

Supplementary Figure 3. **Factor loadings of the neuropsychological tasks**. Dashed vertical lines indicate factor loading cut-offs (>0.5).

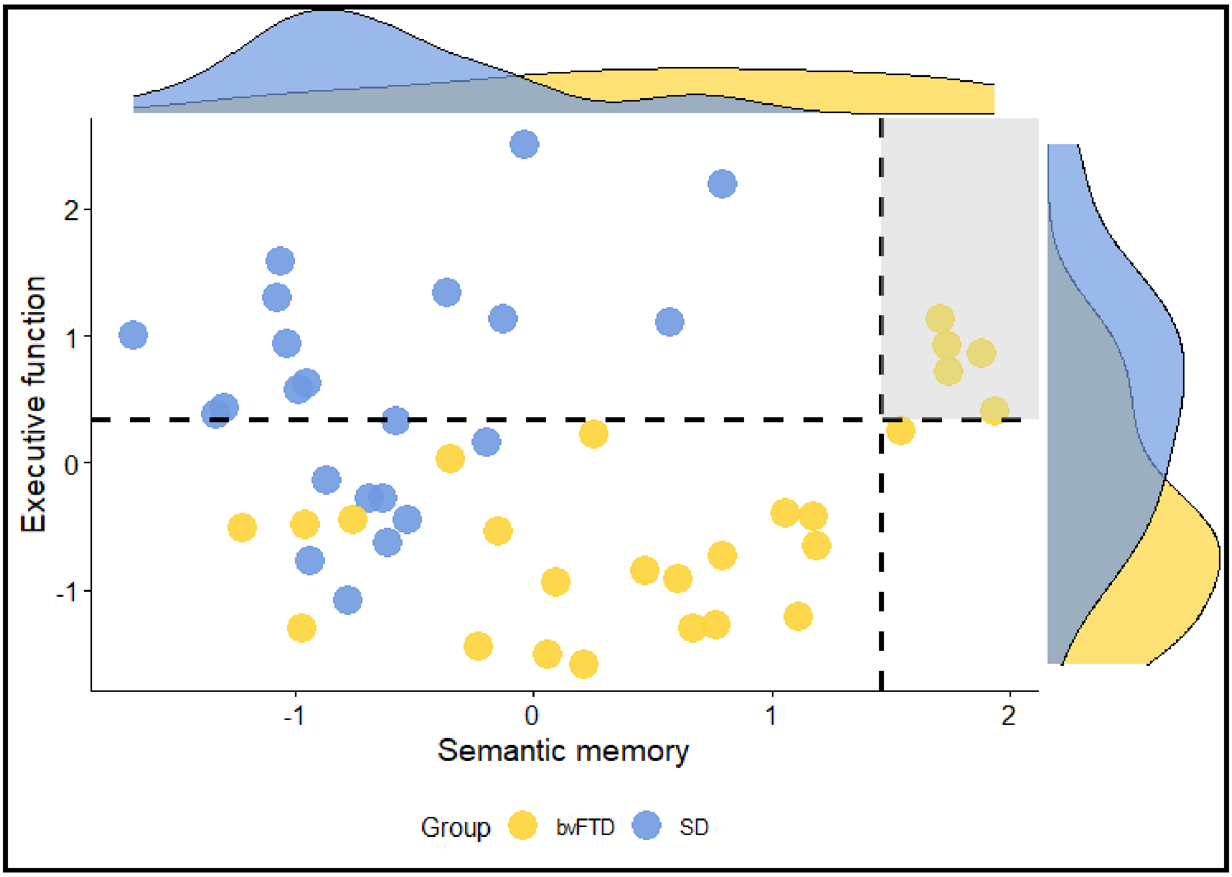

Supplementary Figure 4. Neuropsychology factor scores. Dashed lines indicate the factor score of a control scoring 1.96 standard deviations below the control average on each task and the shaded region shows the region of preserved performance.
